# Semaglutide treatment in hypothalamic obesity: Two-Year outcomes on body composition, appetite, and quality of life

**DOI:** 10.1007/s11102-025-01564-7

**Published:** 2025-08-19

**Authors:** Erlend Gjersdal, Frederik Østergaard Klit, Kåre Schmidt Ettrup, Peter Vestergaard, Eigil Husted Nielsen, Kristian Nilsson Vistisen, Hermann L. Müller, Dorte Melgaard, Jakob Dal

**Affiliations:** 1 Department of Endocrinology, Aalborg University Hospital, Aalborg, Denmark; 2 Steno Diabetes Center North Denmark, Aalborg, Denmark; 3 Department of Clinical Medicine, Aalborg University Hospital, Aalborg, Denmark; 4 Department of Neurosurgery, Aalborg University Hospital, Aalborg, Denmark; 5 Department of Pediatrics and Pediatric Hematology/Oncology, University Children’s Hospital, Carl von Ossietzky Universität Oldenburg, Klinikum Oldenburg AöR, 26133 Oldenburg, Germany; 6 Department of Emergency Medicine and Trauma Center, Aalborg University Hospital, EMRUn, Aalborg, Denmark

**Keywords:** Hypothalamic obesity, Craniopharyngioma, GLP-1RA, Semaglutide, Hyperphagia

## Abstract

**Purpose:**

Hypothalamic obesity is a severe complication of craniopharyngioma, marked by hyperphagia and rapid weight gain. Glucagon-like peptide-1 receptor agonists such as semaglutide have shown promising effects on weight reduction, but long-term data on weight outcomes, metabolic parameters and quality-of-life remain limited.

**Methods:**

Four female patients with hypothalamic obesity following craniopharyngioma treatment received semaglutide for 24 months. Assessments included DXA scans, metabolic biomarkers, and The Three-Factor Eating Questionnaire at baseline, 6, 12, and 24 months. Interviews at 24 months explored hunger, side effects and quality of life.

**Results:**

After 24 months, median weight loss was 16% (95% CI: 8 to 34%, *p* = 0.004), with maximal loss of 17% at 6 months. Emotional and uncontrolled eating scores (range: 0–100) decreased by 44 (95% CI: − 69 to − 19, *p* = 0.011) and 27 units (95% CI: − 63 to 9, *p* = 0.097), respectively. Interviews revealed reduced hunger, improved self-confidence, less isolation, and higher productivity. Treatment was well tolerated; side effects were mainly mild GI symptoms. Fat and lean mass decreased by 10% (95% CI: 2 to 44%, *p* = 0.016) and 19% (95% CI: 14 to 26%, *p* < 0.001), respectively, with stable bone mineral content. Hemoglobin A1c and LDL cholesterol declined by 6.4 mmol/mol (95% CI: 2.3 to 9.9, *p* = 0.016) and 0.5 mmol/L (95% CI: 0.3 to 0.7, *p* = 0.012).

**Conclusion:**

Semaglutide is a safe treatment that led to long-term sustained improvements in eating behavior, weight control, and improved metabolic health. Patients reported an improved quality of life, which persisted after body weight stabilization.

## Introduction

Hypothalamic obesity (HO) is characterized by abnormal weight gain resulting from hypothalamic dysfunction due to various etiologies, including structural injury (e.g., from craniopharyngiomas, other local tumors, surgery, radiation, or trauma) or genetic disorders such as Prader-Willi syndrome. The disruption of hypothalamic neuroendocrine pathways regulating hunger, satiety, and energy balance often results in hyperphagia and unfavourable eating behaviour, where increased snacking, nightly eating and food preference alterations have all been described [1–5]. This, in combination with reduced energy expenditure [6], leads to rapid fat accumulation, insulin resistance, and severe obesity, substantially increasing cardiometabolic morbidity, mortality, and significantly reducing quality of life [7–9].

Historically, hypothalamic obesity has been refractory to conventional interventions, including dietary modification and physical activity [7, 10]. Pharmacological treatments have generally shown limited efficacy [11]. While glucagon-like peptide-1 receptor agonists (GLP-1RA) have demonstrated success in management of primary obesity [12], studies evaluating their effectiveness in HO have produced mixed results, with trials of exenatide and liraglutide failing to consistently achieve meaningful weight reduction or metabolic improvements [13–18]. Bariatric surgery has been shown to produce significant weight loss, but markedly less so than in patients with primary obesity [19].

Recently, semaglutide, a newer and more potent GLP-1RA has emerged as a promising therapeutic candidate for HO, but many questions remain unanswered or need further investigation [10]. Semaglutide has a longer half-life and greater weight loss efficacy than first-generation GLP-1RAs and it has been shown to engage distinct CNS regions more robustly than liraglutide [20, 21]. Furthermore, it is documented that patients experience significantly reduced hunger and improved quality of life while taking semaglutide [22]. The case report published by Sciacovelli et al. in 2023 described significant weight loss of 25% (31 kg) in 6 months, in 1 male patient with HO treated with semaglutide [23]. We were the first to show an improvement in eating behaviour, body composition and metabolic markers with semaglutide treatment during the first six months [24], with our patients losing an average of 17% (20.2 kg) of their body weight. Weight loss was also reported shortly afterwards in a larger cohort of 26 patients with an average reduction of 10.4% (12.5 kg) over 24 months [25]. A recently published case-control study, including 10 patients in the semaglutide treatment arm, reported that 90% achieved a weight loss of more than 5% after 6 months of treatment [26]. The first case report of tirzepatide used to treat hypothalamic obesity was also published only recently, showing a 6.4% (9 kg) weight loss within 4 months [27].

The long-term effects of semaglutide in HO on hunger suppression and weight loss remain uncertain, as does its potential impact on bone mass and muscle function. This is particularly relevant given the growing recognition of HO as a chronic, relapsing condition, underscoring the need to validate initial positive findings across time and diverse patient populations [10]. Beyond its weight-reducing and metabolic effects, semaglutide may also enhance quality of life in domains such as physical functioning, mobility, and energy [12], however, these potential benefits remain to be systematically evaluated.

To address these knowledge gaps, this study provides longer-term follow-up data on the safety, tolerability, and efficacy of semaglutide treatment in patients with HO, alongside a systematic evaluation of patient-reported experiences through qualitative assessments.

## Methods and materials

Four female patients (mean age 48 years, range 22–69) with HO were recruited from the endocrinology outpatient clinic at Aalborg University Hospital and treated with semaglutide for two years. No other weight loss medication, including setmelanotide, was prescribed during treatment with semaglutide, and no patients had previously been treated with medications indicated for weight loss. Patients were diagnosed with craniopharyngioma (mean age at diagnosis 17.5 years, range 6–49) and subsequently developed (pan)hypopituitarism and HO after treatment involving surgery (1–4 interventions) and radiation therapy. Inclusion criteria, baseline characteristics, prescribed dietary advice (balanced, calorie restricted diet, with a suggested meal plan) and additional details of this study population have previously been published for this cohort [24]. Written informed consent for publication of their clinical details and/or clinical images was obtained from the patients and data were managed in anonymized form.

Semaglutide doses were titrated over the first six months to 1.7-2.4 mg once weekly. Assessments were performed at baseline, 6, 12, and 24 months, including whole-body Dual-energy X-ray Absorptiometry (DXA), body weight, eating behaviour (via the revised 18-item Three-Factor Eating Questionnaire, TFEQ-R18 [28]), and fasting biochemical markers (alanine aminotransferase (ALAT), glycated hemoglobin A1c (HbA1c), triglycerides (TG), low-density lipoprotein (LDL) and high-density lipoprotein (HDL). Dietary counselling was provided at baseline, with adherence reviewed at follow-up nurse visits.

## Results

### Medication dosing and adjustments

At 12 months, two patients were receiving 1.7 mg and two patients 2.4 mg of once-weekly semaglutide treatment. By 24 months, one patient remained on 1.7 mg, while three were receiving 2.4 mg weekly. Although all patients initially reached the maximum dose (2.4 mg), one patient was subsequently reduced to 1.7 mg, due to exacerbation of adverse effects (mild GI symptoms). Levothyroxine doses were reduced in all patients, by a mean of 13.9% (range: 100–350 µg weekly) at follow-up (either at 3 or 6 month follow up). Somatropin doses were increased in two patients (from 0.5 to 0.6 mg, and from 0.7 to 0.8 mg, both at the 12-month visit), remained unchanged in one patient, and was reduced by 0.05 mg in one patient (0.3 to 0.25 mg, 6 month follow up). Hydrocortisone doses remained unchanged throughout the observation period.

### Qualitative interviews

The qualitative component of this study aimed to gain an in-depth understanding of whether semaglutide influenced the patients’ perceived quality of life. A phenomenological-hermeneutic qualitative research design was employed [29]. Four patients were invited to participate in interviews, and none withdrew. Interviews were conducted via telephone by senior researcher D.M., who was not involved in the participants’ clinical care. A semi-structured interview guide was used to explore patients’ psychological, physical, and social experiences related to weight loss with semaglutide. The guide included open-ended questions designed to elicit detailed, reflective responses while minimising interviewer bias. All interviews were audio-recorded and transcribed verbatim with participants’ consent to ensure data accuracy. The transcripts were analysed using Braun and Clarke’s reflective thematic analysis, employing an inductive approach [30]. The analysis was conducted by J.D., E.G., and D.M. For the sake of anonymity, participants are identified by number in all quoted material.

### Statistics

Variables including weight, DXA measurements and TFEQ-R18 scaled score values as well as absolute differences were assumed normally distributed. Blood markers and relative differences were assumed lognormal and transformed accordingly. TFEQ-R18 scaled scores were calculated as (raw score-minimum possible score)/(maximum possible score-minimum possible score ​) × 100. Data were described by means and standard deviations (SD) on the original scale. Differences from baseline to follow-up of lognormal data were back transformed and converted to additive measures by (exp(mean difference) × median_baseline_) - median_baseline_. All comparisons were conducted by paired t-tests. Estimates were reported with 95% confidence intervals (95%CI), and statistical significance was evaluated using an alpha level of 0.05. Data management and statistical calculations were performed in Stata (StataCorp. 2023. Stata Statistical Software: Release 18. College Station, TX: StataCorp LLC).

### Eating behaviour

**Fig. 1:**
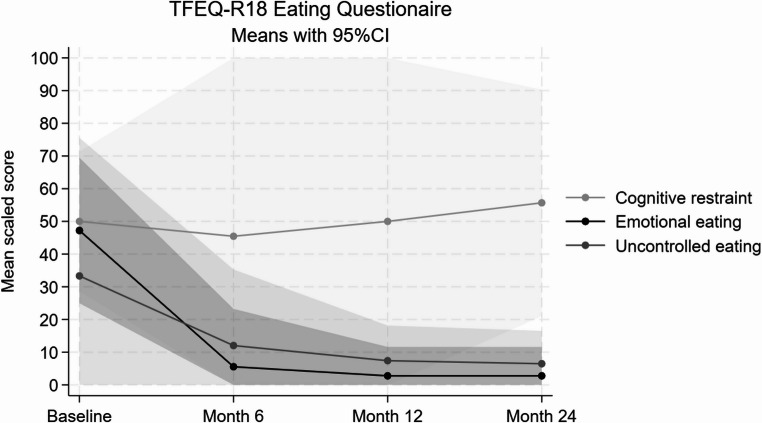
Changes in mean scaled scores from the TFEQ-R18 eating behaviour questionnaire, from baseline to 24 months during semaglutide treatment

Scaled scores of UE and EE according to the TFEQ-R18 questionnaire continued their decline until the 24th month (𝚫 baseline UE: − 27, 95%CI: − 63; 9, *p* = 0.097; EE: − 44, 95%CI: − 69; − 19, *p* = 0.011). Cognitive restraint (CR) scores fluctuated around 50, with no significant changes between baseline and 24 months (95%CI: − 30; 42, *p* = 0.651) (see Fig. 1). The main improvement in eating behavior appeared during the initial months of treatment as previously reported [24].

### Body weight and body composition

**Fig. 2:**
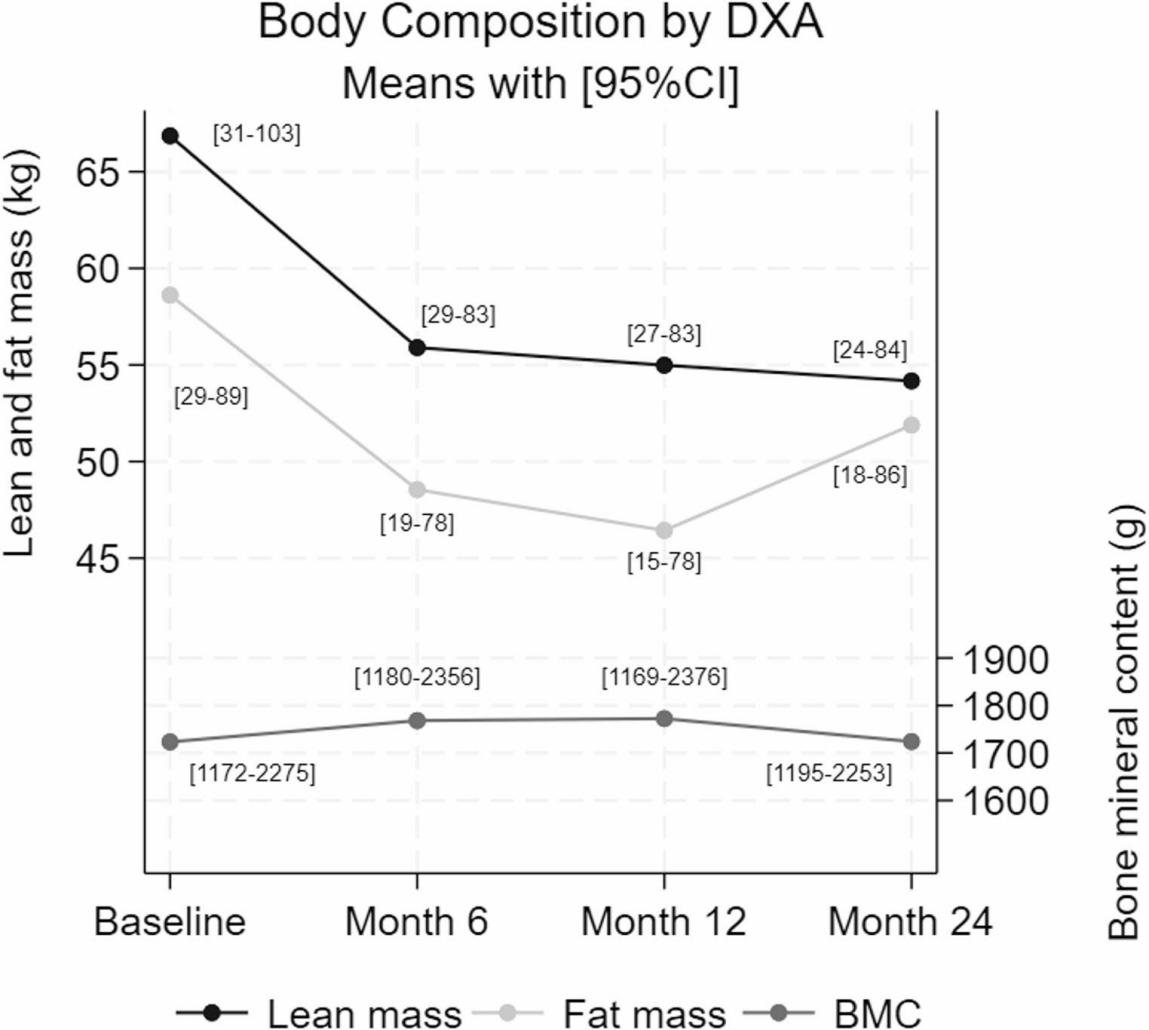
Changes in body composition measured by dual-energy X-ray absorptiometry (DXA) from baseline to 24 months during semaglutide treatment

Body weight and BMI remained reduced at 24 months from baseline (median 16%, 95%CI 8; 34%, *p* = 0.004; mean − 8.0 kg/m2, 95%CI: − 4.0; − 11.9, *p* = 0.008). Most participants sustained weight loss with minor fluctuations related to seasonal events, intermittent treatment interruptions, or intercurrent illness. The maximal average weight loss of 17% was achieved within 6 months, as previously documented [24]. Both fat and lean masses were reduced significantly after 24 months of treatment (median 10%, 95%CI: 2; 44%, *p* = 0.016 and median 19%, 95%CI: 14; 26%, *p* < 0.001, respectively). DXA scans indicated that the sustained weight loss was primarily from lean mass, as fat mass, although initially reduced, increased again after month 6 (median ratio of lean to fat mass reduction 1.90, 95%CI: 0.5; 7.20, *p* = 0.223). From month 12 to 24, median weight increased slightly by 3% (95%CI: 1; 14%, *p* = 0.006) and median fat mass increased by 11% (95%CI: 6; 23% (1–10 kg), *p* = 0.002). Individual weight gains over this period were 3.0, 2.0, 0.8, and 14.0 kg, with corresponding increases in fat mass of 4.6, 4.2, 3.0, and 10.0 kg, respectively. The BMC (composite of arms, ribs, spine, pelvis and legs) was unchanged from baseline to follow-up at 24 months (mean 𝚫 baseline 1 g, 95%CI: − 97; 98, *p* = 0.983) (see Table 1; Fig. 2).

**Table 1:** Changes in body composition from baseline to 24 months during semaglutide treatment. BMC = bone mineral content

Body Composition	Baseline (mean ± SD) [95%CI of SD]	6 Months (𝚫 baseline) [95% CI]	12 Months (𝚫 baseline) [95% CI]	24 Months (𝚫 baseline) [95% CI]
Weight (kg)	126 ± 42 [24–156]	−20 [–26; − 14]	−25 [–45; − 6]	−21 [–32; − 9]
BMI (kg/m²)	48.0 ± 9.4 [5.3–35.1]	−7.9 [–10; − 5.5]	−9.7 [–15.5; − 3.9]	−8.0 [–11.9; − 4.0]
Fat Mass (kg	59 ± 19 [11–70]	−10 [–15; − 6]	−12 [–21; − 4]	−7 [–14; 0]
Lean Mass (kg)	67 ± 23 [13–85]	−11 [–22; 0]	−12 [–23; − 1]	−13 [–20; − 6]
Bone Mineral Content (g)	1723 ± 347 [196–1293]	45 [–21; 110]	49 [–26; 124]	1 [–97; 98]

### Qualitative assessments

During analysis of the interviews after 24 months of semaglutide treatment, five main themes emerged; (1) Hunger and thoughts about food, (2) mobility and musculoskeletal pain, (3) social life, (4) work life and (5) side effects to semaglutide treatment. These themes highlight the participants’ experiences with weight loss after semaglutide treatment and its impact on different aspects of their daily lives.

### Hunger and thoughts about food

Participants reported a significant and lasting reduction in persistent hunger, describing how they no longer felt an overwhelming drive to consume food, starting already shortly after treatment initiation. For some, this change allowed them to regulate portion sizes more effectively and develop a more natural sense of satiety:

*“I no longer have that endless hunger - I was never full before*,* but now I am.” (1)*.

*“I have to say*,* it’s gotten easier to hold back at mealtimes. Before*,* I wouldn’t think twice about going for a second helping*,* but now I find it easier to wait 20–30 minutes after my first plate to see if I’m still hungry.” (2)*.

These findings suggest that semaglutide plays a role in suppressing the feeling of excessive hunger, allowing for more controlled eating behaviours in these individuals with hypothalamic obesity.

### Mobility and pain

Several participants reported experiencing improved mobility and reduced musculoskeletal pain, attributing these changes to their weight loss. Everyday activities, such as bending down or going on walks, became easier:

*“Also*,* for example*,* when I’m tidying up at the kindergarten*,* it’s easier for me to bend down and pick up the markers from the floor.” (3)*.

*“I can tell my knees don’t hurt anymore*,* unlike before. That’s probably because I was carrying more weight back then.” (4)*.

Additionally, some participants noted that they could engage in outdoor activities with greater ease and enjoyment:

*“I really enjoy getting out into nature now*,* whether I’m walking or cycling.” (4)*.

Other changes included increased endurance and a noticeable improvement in breathing:

*“I don’t get out of breath as easily.” (1)*

*“Just being able to breathe - I can actually fill my lungs completely now.” (2)*.

Overall, participants described feeling physically stronger, less fatigued, and more capable of completing daily tasks with greater efficiency.

### Social life

Weight loss and improved well-being had a positive impact on participants’ social lives. Several expressed newfound confidence in public settings and an increased willingness to engage in social interactions:

*“Well*,* I’m glowing - and it’s done wonders for my confidence. It’s made it so much easier to go out and meet new people*,* whereas before*,* I tended to hold back a bit.” (4)*.

Some participants also mentioned that they were no longer isolating themselves at home:

*“I was brave enough to go to the folk high school as well.” (3)*.

*“…before*,* I used to hide away within my own four walls*,* but now I go out*,* meet other people*,* and gain new experiences. My social network has grown*,* and that has had a positive impact on my confidence.” (4)*.

This increase in self-confidence also led to a greater interest in personal appearance and clothing choices:

*I also feel braver about going into stores I wouldn’t have visited before - browsing clothes*,* trying things on*,* and even asking for the staff’s opinion. Like*,* ‘What do you think?’.” (4)*.

*“I’m so proud that I can just go to the supermarket now and buy clothes. I actually did it… I bought a blouse just before Christmas…” (3)*.

### Work life

Participants reported positive changes in their work life, particularly in terms of increased energy levels and productivity:

*“Before*,* I used to feel tired after cleaning - and at work too. But now it takes half the time because it’s so much easier to get through. I can really feel my energy blooming. I’m not exhausted after a workday like I used to be. Before*,* I was completely drained and couldn’t do anything at home after working six or eight hours.” (4)*.

For some, weight loss also led to a greater willingness to seek new job opportunities:

*“I’m not working full-time right now. At the moment*,* I’m working as a part-time substitute*,* but I’m looking for a permanent position. I feel more confident going to interviews and presenting myself as I am now.” (4)*.

These findings suggest that changes in hunger and weight loss not only improved participants’ physical health, but also enhanced their professional self-esteem and career aspirations.

### Side effects

Local irritation in the area of the injection site and hair loss was reported by one patient each, but gastrointestinal symptoms were the most common side effect:

*“I have really*,* really hard stools - it’s very painful.” (4)*.

*“It’s like my digestion just stops*,* as if the food won’t go do down my food pipe properly” (4)*.

*“When I eat fatty food*,* I have to go to the toilet shortly after.” (2)*.

*“I felt unwell when I ate fatty foods - it almost always made me vomit and gave me a headache - but it’s completely fine now.” (1)*.

## Biomarkers of metabolism

Metabolic profile overall showed a slight tendency of improvement (see Table 2). Specifically, HbA1c was reduced by 6.4 mmol/mol (95%CI: − 2.3; − 9.9, *p* = 0.016) and LDL by 0.5 mmol/L (95%CI: − 0.3; − 0.7, *p* = 0.012). Likewise, TG showed a tendency over the follow-up period towards a reduction of 1.0 mmol/L (95%CI: −1.2; − 1.8, *p* = 0.129), significant at 6 (*p* = 0.046) and 12 (*p* = 0.041) months, but non-significant at 24 months where 1 measurement was lost. ALAT and HDL remained largely unchanged.

**Table 2:** Changes in metabolic biomarkers from baseline to 24 months during semaglutide treatment. ALAT = alanine aminotransferase, HbA1c = glycated hemoglobin A1c, tg = triglycerides, LDL = low-density lipoprotein, HDL = high-density lipoprotein

	Baseline mean ×/÷ SD [95%CI of SD]	6 months (𝚫 baseline) [95% CI]	12 months (𝚫 baseline) [95% CI]	24 months (𝚫 baseline) [95% CI]
ALAT (U/L)	34.1 ×/÷ 63.3% [32.0-523.8%]	−1.3 [–16.; 27.6]	−7.7 [–23.0; 28.7]	5.8 [–21.3; 89.6]
HbA1c (mmol/mol)	38.1 ×/÷ 11.6% [6.4–50.8%]	−4.1 [–7.3; −0.7]	−6.7 [–9.3; −3.7]	−6.4 [–9.9; −2.3]
TG (mmol/L)	2.2 ×/÷ 36.9% [19.5-222.8%]	−0.9 [–1.4; 0.0]	−1.0 [–1.6; − 0.1]	−1.0 [–1.8; 1.2]
LDL (mmol/L)	2.5 ×/÷ 30.3% [16.2-168.2%]	−0.3 [–0.9; 0.7]	−0.4 [–0.9; 0.4]	−0.5 [–0.7; − 0.3]
HDL (mmol/L)	1.1 ×/÷ 17.5% [9.6–82.7%]	−0.2 [–0.2; − 0.1]	−0.0 [–0.1; 0.0]	0.1 [–0.3; 0.6]

## Discussion

This case series provides a unique long-term evaluation of semaglutide treatment in patients with hypothalamic obesity (HO) over a 24-month period, with regular outpatient follow-up and a focus on physical activity and healthy dietary habits. We incorporated comprehensive assessments of body composition, metabolic health, eating behavior, and patient-reported quality of life.

A clinically significant and sustained weight loss of median 16% (mean: 21 kg) was achieved at 24 months which is in line with other cohorts [25]. This outcome was primarily attributed to improvements in eating behavior, which became evident shortly after treatment initiation [24] and persisted throughout the follow-up period. These changes were captured through both the TFEQ-R18 questionnaire and patient interviews. The “Uncontrolled Eating” (UE) subscale showed a marked reduction, indicating improved control over food intake, while the “Emotional Eating” (EE) subscale suggested a diminished tendency to eat in response to negative emotions. These reductions in UE and EE reflect the appetite-suppressing effects of semaglutide, as previously reported in individuals with primary obesity or type 2 diabetes mellitus [31–33].

However, a modest median weight regain (3%) was observed between months 12 and 24, primarily driven by an increase in fat mass. This regain was largely attributable to one participant who struggled to maintain dietary and exercise habits, although overall improvements in eating behavior persisted. In this patient the treatment was temporarily paused, once for one month between months 13 and 14, and again for one week at month 15, due to a busy schedule and time away on vacation. A slight increase in fat mass was observed across all participants, suggesting that the long-term effects of semaglutide in individuals with hypothalamic obesity warrant further investigation. We speculate that potential contributors to weight regain in our cohort may include temporary interruptions in semaglutide treatment (due to delayed access to subsidized medication), intercurrent illness, or psychosocial stress. Additional contributors may involve reduced physical activity and suboptimal adherence to a healthy diet. These findings underscore the importance of ongoing support and regular monitoring to promote treatment adherence in this patient population. An oral formulation of semaglutide is available, and may offer a more accessible option for patients who face challenges with maintaining injectable treatment regimens, but it has not been studied in patients with hypothalamic obesity.

Lean body mass declined mainly during the first six months of treatment and then stabilized. The literature reports substantial variation in changes in lean mass during semaglutide treatment. A recent meta-analysis reported changes in lean body mass ranging from preservation of lean mass with a general improvement in the lean-to-fat mass ratio reduction, to a reduction in lean mass accounting for up to 40% of total weight lost [34]. We observed a 19% (13 kg) reduction, however, our patients reported improved physical capacity throughout the follow-up period, suggesting preserved muscle function. Bone mass content remained stable during weight loss, contrasting with findings from recent studies in older adults with osteopenia, which demonstrated reductions in lumbar spine and hip BMD following semaglutide treatment [35]. Other studies evaluating GLP-1RA effects on bone health in adults have shown maintenance or even improvements in bone mass, consistent with our observations [36–41]. The preservation of bone health in our cohort may be partly attributed to increased physical activity overall, as subjectively reported by all patients in the interviews. Unfortunately, we did not assess changes in muscle strength, which might have supported this claim, if increased or preserved.

We observed marked improvements in glycemic control, with reductions in HbA1c and improvement in dyslipidemia, reflecting an overall healthier cardiometabolic profile and a likely reduction in long-term cardiovascular risk. These outcomes are consistent with previous findings in individuals with obesity treated with semaglutide [42, 43].

In the interviews, patients reported marked improvements in several key aspects of their quality of life, including improved physical functioning and enhanced social and work life. Social functioning was a particularly prominent area of improvement, consistent with existing evidence which indicates that obesity adversely impacts this domain [44]. Participants attributed increased social engagement to weight loss, echoing previous findings [45]. They also emphasized the value of being able to shop in regular clothing stores - an improvement strongly associated with an enhanced quality of life. The psychological significance of this change is supported by prior research linking clothing size to overall life satisfaction [46].

In terms of work life, participants described greater ability to manage physically demanding tasks, matching data showing an inverse relationship between BMI and work capacity [47]. One participant had even secured permanent part-time employment (30 h/week) following the interviews, reflecting meaningful vocational improvement. Enhanced mobility and reduced joint pain, as reported by participants, further underscored the physical benefits of treatment and aligned with earlier observations [22]. Patients noted only common side effects, all of which are well described in existing literature [48], and none were severe enough to result in treatment discontinuation.

In conclusion, the observed improvements in eating behavior, body composition, metabolic risk profile, and quality of life suggest that semaglutide treatment in patients with hypothalamic obesity provides benefits that extend beyond weight loss. The treatment was well tolerated, with only minor and transient gastrointestinal side effects, and no participants discontinued therapy. Most effects became apparent shortly after treatment initiation and either remained stable or continued to improve over time. However, partial regain of fat mass and body weight was observed between months 12 and 24, underscoring the need for larger studies with longer follow-up periods. We also observed positive impacts on work and social life, reduction in excessive hunger, and improvements in physical function following weight stabilization. However, ensuring continued adherence to treatment remains critical in this patient population. Findings from previous withdrawal studies in patients with primary obesity treated with GLP-1RA along with our observations during unintentional treatment interruptions, indicate that GLP-1RA therapy in HO may need to be considered a long-term, potentially lifelong, intervention.

## Data Availability

No datasets were generated or analysed during the current study.
